# RUNX3 Inhibits the Invasion and Metastasis of Human Colon Cancer HT-29 Cells by Upregulating MMP-2/9

**DOI:** 10.1155/2020/5978131

**Published:** 2020-02-27

**Authors:** Jun Xue, Xueliang Wu, Ming Qu, Fei Guo, Lei Han, Guangyuan Sun, Zelong Yuan, Shuang Fan, Tian Li

**Affiliations:** ^1^Department of General Surgery, First Affiliated Hospital of Hebei North University, Zhangjiakou 075000, China; ^2^School of Basic Medicine, The Fourth Military Medical University, 169 Changle West Road, Xi'an 710032, China

## Abstract

**Objective:**

To investigate the effect of Runt-associated transcription factor 3 (RUNX3) on the invasion and metastasis of human colon cancer HT-29 cells and to preliminarily explore the mechanism of its anticancer effect.

**Methods:**

The RUNX3 plasmid vector was transfected into human colon cancer HT-29 cells by liposome-mediated transfection, while the empty vector and the blank group were used as the control group. After Geneticin (G418) screening, HT-29 cells with stable expression of RUNX3 gene were obtained. The expressions of mRNA and proteins of RUNX3 and metalloproteinases (MMP)-2/9 were detected by reverse transcription-polymerase chain reaction (RT-PCR) and western blot. Cell proliferation was determined by MTT assay. The effect of RUNX3 on invasion and metastasis of HT-29 cells was evaluated by scratch injury assay, Transwell chamber, and Matrigel invasion model.

**Results:**

RUNX3 was expressed stably in HT-29 cells after transfection. The expressions of RUNX3 mRNA and proteins in the experimental group were significantly higher than those in the blank/empty vector groups. Meanwhile, the expressions of MMP-2/9 mRNA and proteins in the observation group were significantly lower than those in the blank group and the empty vector group. The proliferation and migration ability in the experimental group was significantly lower than blank/empty vector groups from the third day. Transwell chamber experiment and Matrigel invasion assay showed that the number of Transwell cells was decreased significantly than blank/empty vector groups, but no difference was found between the blank group and the empty vector group.

**Conclusion:**

RUNX3 can inhibit the invasion and metastasis of human colon cancer HT-29 cells, and the mechanism may be related to decreased expression of MMP-2 and MMP-9.

## 1. Introduction

Cancer, cardiocerebrovascular disease, and nervous system disease are three major killers among humans worldwide [1, 2]. According to the Global Cancer Statistics 2018, over 1.8 million new colorectal cancer cases and 881,000 deaths are estimated to occur in 2018. Colorectal cancer ranks third in terms of incidence whereas second in mortality [3, 4]. Colorectal cancer is one of the most common solid tumors worldwide, and the incidence and mortality of colorectal cancer ranks the third and fifth, respectively, in China. It is seriously threatening national health and safety. Recently, although the application of comprehensive treatments such as surgery, radiation therapy, chemotherapy, biological therapy, and immunotherapy has improved the curative effect and prolonged survival, some patients fail to meet the best period of treatment due to difficulties in early screening/diagnosis [5, 6], and there still remains a large gap between China and Europe/US. Therefore, clarifying the pathogenesis from the perspective of molecular biology and seeking markers with high sensitivity/specificity have become the research focus of colorectal cancer [7, 8].

Runt-related transcription factor 3 (RUNX3) is a newly discovered tumor suppressor gene, which regulates cell proliferation, growth, and apoptosis via transforming growth factor *β* (TGF-*β*) and Wnt pathways [8, 9]. MMP degrades the extracellular matrix and basement membrane and plays significant roles in promoting tumor invasion and metastasis. MMP proteolytically activates or degrades a variety of nonmatrix substrates, including cytokines and chemokines, exerting a regulatory function in inflammation and immunity [10, 11]. MMP-2/9 is not only involved in a variety of pathophysiological processes, but also play a promoting role in tumor invasion and angiogenesis. A large number of studies confirmed that the abnormally high expression of MMP-2/9 is closely related to the variety of metastasis and poor prognosis of cancer [12, 13]. Our previous studies had confirmed that downregulation of RUNX3 expression was closely related to the occurrence and development of colorectal cancer, whereas the specific mechanisms were not reported in detail [14].

In this manuscript, the eukaryotic expression vector of RUNX3 is introduced into colon cancer HT-29 cells [15]. We investigate the changes in the invasion and metastasis ability of the cells *in vitro* and preliminarily explore the internal mechanisms.

## 2. Materials and Methods

### 2.1. Reagents

Recombinant plasmid pcDNA3.1/V5-His-TOPO/RUNX3 and liposome transfection reagent Lipofectamine™ 2000 were bought from Invitrogen. Total RNA extraction reagents and RIPA reagents were bought from Thermo Fisher Scientific (USA). The PCR reagent kit was bought from Promega. The SDS-PAGE standard indicator and the reverse transcription kit were bought from Fermentas. The ECL reagent was purchased from Pierce. A Transwell chamber was bought from Coster. Matrigel was purchased from BioRad.

### 2.2. Cell Culture

Human colon cancer HT-29 cells were stored in the cell bank of the scientific research center of Hebei North University. HT-29 cells were normally cultivated in the RPMI-1640 medium (Solarbio Beijing, China), 10% fetal bovine serum (FBS, Gibco), 100 *μ*g/mL streptomycin, and 100 U/mL penicillin (Gibco) in a 5% CO_2_ atmosphere at 37°C. HT-29 cells were subcultured by trypsin (Solarbio Beijing, China) when the cell density reaches 80%–90%.

### 2.3. Recombinant Plasmid Transfection

Cells in the logarithmic phase were chosen before treatment and were transfected in 24-well plates, where cell growth was 70%–80% confluent, by Lipofectamine transfection™ 2000 (Thermo Fisher Scientific, Waltham, USA). Meanwhile, pcDNA 3.1/v5-his-topo plasmid (Thermo Fisher Scientific, Waltham, USA) without RUNX3 cDNA was used as the empty vector group, and HT-29 cells without transfection was used as the blank group. After G418 screening, stable RUNX3 expression was obtained as the observation group.

### 2.4. MTT Assay

Cells in the logarithmic phase were normally digested and inoculated in a 96-well plate. 200 *μ*L suspension containing 1 × 10 4 cells was inoculated in each well. 20 *μ*L thiazolyl blue (MTT) (*μ*g/*μ*L) was added into each well. Cell lines were surveyed in 3 parallel wells of 3 random types of cells. Cells were inoculated in the medium in 5% CO_2_ at 37°C for 4 h. Supernatant was discarded. 150 *μ*L DMSO was added to every well. Cells were shaken for 10 min for complete crystallization. The 492 nm value (OD value) was surveyed on the ELISA survey meter for successive 7 days. Growth curves were drawn.

### 2.5. Wound Healing Assay

Cells in logarithmic growth phase of the three groups were inoculated into a 6-well culture plate at concentrations of 5 × 105 cells/ml per well, two duplicated wells were set for each group, and cells were cultured in a culture box with 5% CO_2_ at 37°C. The monolayer cell was horizontally scratched in each well with the head of a 200-*μ*L pipette in complete confluence cells. Wound healing model cells were routinely cultured, washed with PBS, and placed in culture medium for 24 hours, and cells were observed and photographs were taken.

### 2.6. Cell Migration and Invasion Ability

Three groups of cells in the logarithmic phase were normally digested and washed by the RPMI1640 medium without fetal calf serums for cell suspension. Three groups of 200 *μ*L cell suspension were added into the upper chamber of the Transwell chamber. 600 *μ*L of the aforementioned medium was added into the lower chamber and cultivated for 24 h. The Transwell chamber was taken out, and the remaining cells were wiped away with a cotton swab. Crystal valet staining was carried out and the number of cells through the membrane as migrating cells were counted under the microscope. Matrigel matrix was laid on the upper polycarbonate membrane of the Transwell chamber. The rest of the procedures were the same as aforementioned. The number of cells through Matrigel matrix was used to evaluate the invasion ability of cells.

### 2.7. RNA Isolation and RT-PCR

RNA was isolated from HT-29 cells by the total RNA extraction kit (Solarbio, Beijing, China) according to the manufacturer's protocol. The quality and quantity of RNA were measured by NanoDrop 1000 (Thermo Scientific, Waltham, USA). Each mRNA expression was normalized to GAPDH mRNA expression using the comparative cycle threshold method. Identity and purity of the amplified product were checked by analyzing the melting curve plotted at the end of amplification.

RT-PCR was performed with the RUNX3 forward primer: 5′-GAGTTTCACCCTGACCATCACTGTG-3′, the reverse primer: 5′-GCCCATCACTGGTCTTGAAGGTTGT-3′; the MMP-2 forward primer: 5′-ACCTGGATGCCGTCGTGGAC-3′, the reverse primer: 5′-TGTGGCAGCACCAGGGCAGC-3′; the MMP-9 forward primer: 5′-TGGAGTCACTGTACACCCTC-3′, the reverse primer: 5′-CGGACATCCGCTAAACAGGT-3′; and the GADPH forward primer: 5′-ACGACCACTTTGTCAAGCTC-3′, the reverse primer: 5′-TCTTCCTCTTGTGCTCTTGC-3′. The RT reaction system included (total reaction volume 25 *μ*L) 5 × PCR buffer 4 *μ*L, random primer 1 *μ*L, RNA enzyme inhibitor 1 *μ*L, dNTP 2 *μ*L, reverse transcription enzyme 1 *μ*L, and DEPC-treated water. The reaction conditions were predenaturation at 95°C for 30 s, denaturation at 95°C for 5 s, annealing at 58°C for 30 s, and extension at 72°C for 15 s (a total of 40 cycles). Results were obtained in ultraviolet rays, and RUNX3/GAPDH and MMP-2/9/GAPDH were used for quantitative determination.

### 2.8. Western Blot

Total protein was extracted by RIPA buffer (Solarbio, Beijing, China). 50 *μ*g protein was put into 5 × loading buffer (4 : 1), 100°C water bath for 10 min, and SDS-PAGE electrophoresis and transferred to polyvinylidene fluoride (Millipore, Darmstadt, Germany). PVDF was blocked by 5% nonfat milk. Specific primary antibody was added (RUNX3 and MMP2/9: 1:1500; GAPDH: 1 : 150) (CST Signaling Technology, Danvers, USA) and incubated at 4°C overnight. After washing with TBST, second antibody (1 : 1500) was incubated at 37°C for 1 h. After washing with TBST, ECL (Millipore, Darmstadt, Germany) was applied, and RUNX3/GAPDH and MMP2/9/GAPDH were used for quantitative determination.

### 2.9. Statistical Analysis

SPSS17.0 statistic software was used. *x* ± *s* was used as measurement data. Single factorial variance analysis was adopted for comparison among groups. Dunnett's test was adopted for comparison between two groups. *P* < 0.05 was regarded as having statistic significant difference.

## 3. Results

### 3.1. Expression of RUNX3 and MMP-2/9 by RT-PCR

**Figure 1:**
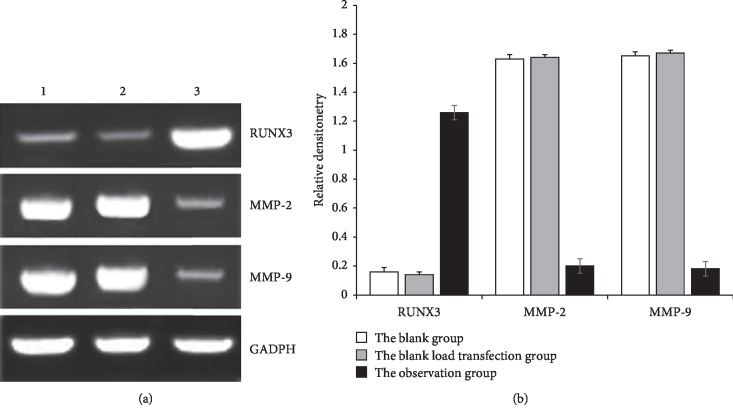
Expression of RUNX3 and MMP-2/9 in different HT-29 cells detected by RT-PCR. 1: The blank group; 2: The blank load transfection group; 3: The observation group (the grouping is the same in Figures 2, 4, and 5).

As shown in Figure 1, in the observation group, the expression level of RUNX3mRNA was 1.26 ± 0.05, higher than that of the blank group (0.16 ± 0.03) and that of empty vector group (0.14 ± 0.02), and there was significant statistical differences (*P* < 0.01). In the observation group, the expression level of MMP-2/9 mRNA was 0.20 ± 0.03/0.18 ± 0.02, lower than that of the blank group (1.63 ± 0.07)/(1.67 ± 0.06) and that of empty vector group (1.64 ± 0.06)/(1.65 ± 0.05), and there was significant statistical differences (*P* < 0.01).

### 3.2. Protein Expression of RUNX3 and MMP-2/9 by Western Blot

**Figure 2:**
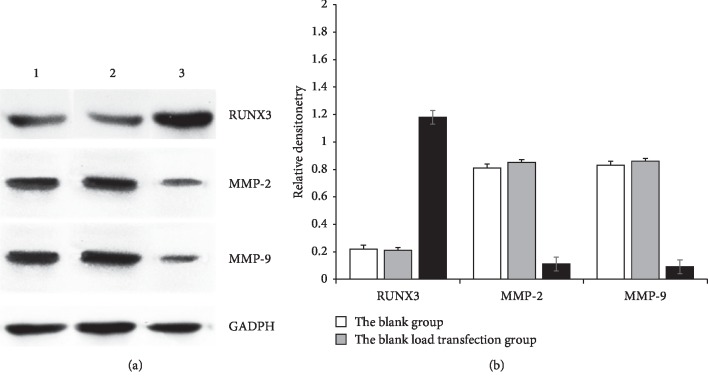
Expression of RUNX3 and MMP-2/9 in different HT-29 cells detected by western blot.

The protein expression level of RUNX3 protein in the observation group was 1.18 ± 0.04, higher than that of the blank group (0.22 ± 0.03) and that of empty vector group (0.21 ± 0.02), and there were significant statistical differences (*P* < 0.01, Figure 2). In the observation group, the protein expression level of MMP-2/9 protein was 0.11 ± 0.01/0.09 ± 0.01, lower than that of blank group (0.81 ± 0.03)/(0.83 ± 0.04) and that of empty vector group (0.85 ± 0.04)/(0.86 ± 0.05), and there was significant statistical differences (*P* < 0.01, Figure 2).

### 3.3. Cell Proliferation Ability by MTT Assay

**Figure 3:**
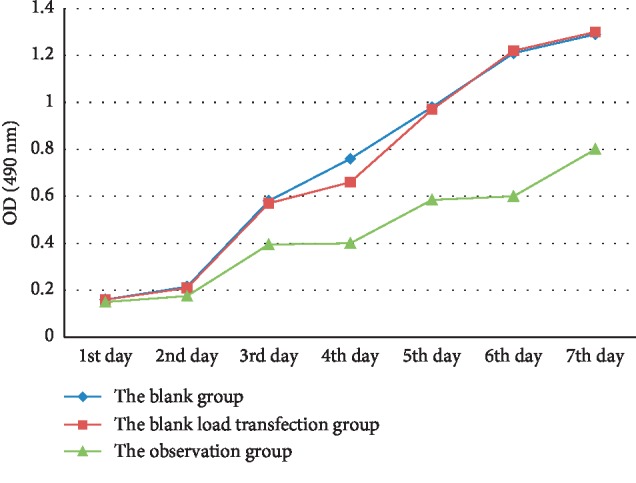
Proliferation curves of different HT-29 cells detected by MTT assay.

As shown in Figure 3, OD values of cells in HT-29 cell lines did not differ significantly, and growth curves basically overlapped after cultivation for successive 1 or 2 d; since the third day, proliferation of observation groups began to decrease; since the fourth day, inhibition of growth in observation groups was obvious, and the OD value significantly decreased compared to the blank groups and the empty vector groups. Growth curves of cells in corresponding blank groups and empty vector groups were basically close to each other, and there is not much difference in the proliferation ability.

### 3.4. Scratch Damage Test

**Figure 4:**
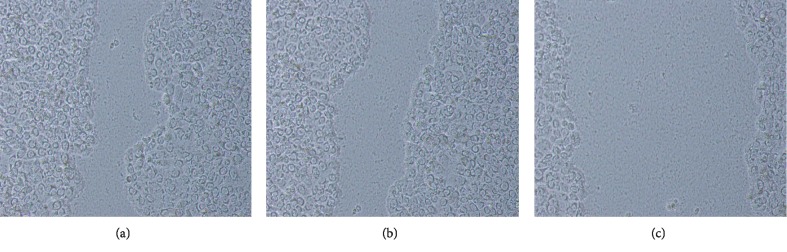
Scratch damage test of HT-29 cells.

The cells in the observation group migrated to the center slowly, and the repair of the defect was slow (Figure 4). The cells in the blank group and the empty vector group migrated to the scratch significantly faster than those in the observation group, but there was no significant difference in the migration speed between the blank group and the empty vector group.

### 3.5. Cell Migration and Invasion Ability

**Figure 5:**
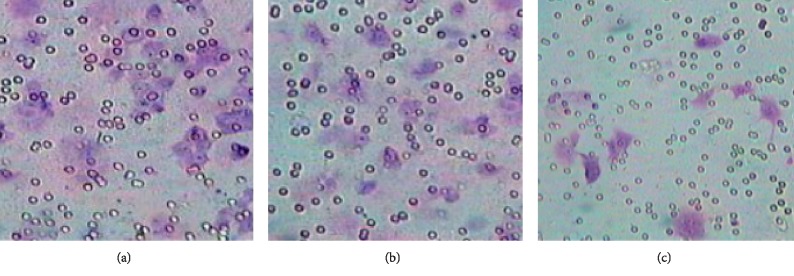
Detection cell invasion ability of different HT-29 cells.

As shown in Figure 5 and Table 1, Transwell chamber migration and Matrigel invasion experiments showed that numbers of cells through the membrane/matrix in the observation groups significantly decreased compared to corresponding blank groups and empty vector groups (*P* < 0.001). There was not much difference in numbers of cells through the membrane/matrix between the blank groups and the empty vector groups (*P* > 0.05).

## 4. Discussion

RUNX3, a member of human Runt-related transcription factors, was firstly reported by Levanon in 1994 [16]. It is a pivotal endogenous gene that plays an important role in cell growth, apoptosis, differentiation, and tumorigenesis. The RUNX3 gene is located on chromosome 1p36.1 and has six exons and 1290 bp open reading frame. There are two kinds of variant shearing bodies in length. The upstream of COS region contains P1 and P2 promoters that are responsible for the transcriptional regulation of RUNX3 and are mainly manipulated by P2. Because the P2 promoter is located before Exon 2, the GC content is as high as 64%, and there is a 4.2 kb highly conserved cytosine-phosphate-guanine (CpG) island around the P2 promoter, while the CpG island has the characteristics of GC promoter. Thus, P2 is more likely to be methylated [17, 18]. RUNX3 protein is a heterodimer composed of α-and *β*-subunits that includes 415 amino acid residues with a molecular weight of about 44 kD. The α-subunit contains a Runt domain (RD) conserved domain which is composed of 128 amino acids, located at the end of the amino group. RD contains a S-shaped immunoglobulin folding that mediates the binding of RD to the target DNA and the relationship between core binding factors and proteins. The *β*-subunit is composed of 134 amino acid residues, which can enhance the binding capacity of RD to the target DNA and maintain its normal regulation and control function [19]. RUNX3 can directly bind to the effector molecule Smad3 of transforming growth factor *β*, a signal pathway to participate in the process of cell growth inhibition [20]. RUNX3 can also form complexes with transcription factor 4 (TCF4) and B-catenin, the key effectors of the Wnt pathway, to inhibit the binding of TCF4-*β*-catenin to target DNA, thus weakening the transcription of target genes [21]. In addition, RUNX3 is involved in the regulation of epithelial-mesenchymal transition (EMT) by directly regulating the transcription of blocking protein-1, thus inhibiting tumor invasion and metastasis by protecting nesting apoptosis [22].

MMP-2 can break the degradation balance of matrix, induce cancer cells to penetrate the barrier formed by extracellular matrix and basement membrane, and reduce the adhesion between cells, so that cancer cells can infiltrate, invade, and metastasize to distant organs in surrounding tissues [23, 24]. In the process of degradation, large amounts of vascular endothelial growth factors stored in the extracellular matrix can be released to induce tumor angiogenesis and provide the necessary nutritional basis and pathway for tumor cell invasion [25]. MMP-9, also known as Gelatinase B, can degrade and destroy extracellular matrix and basement membrane near the surface of tumor cells, release a large number of growth promoting factors, and make tumor cells invade the surrounding tissues along the missing basement membrane, promoting the invasion and metastasis of tumor cells [26–28].

In this study, a RUNX3 eukaryotic expression vector was introduced into colon cancer HT-29 cells, and the expression of RUNX3 mRNA and protein were detected by RT-PCR and western blot, taking advantage of its high specificity and sensitivity. The study demonstrated that the expression of RUNX3 mRNA and protein in the observation group were significantly higher than the blank group and empty vector group, whereas the MMP-2/9 mRNA and protein expression in the observation group were significantly lower than those in the blank group and empty vector group, suggesting that the high expression of RUNX3 can inhibit the expression of MMP-2/9 in human colon cancer HT-29 cells to inhibit proliferation, invasion, and metastasis of tumor cell. Some studies also revealed that RUNX3 overexpression inhibited CRC cell migration and invasion. In contrast, knockdown of RUNX3 reduced the inhibition of migration and invasion of colorectal cells [29].

Furthermore, MTT test results showed that the proliferation in the observation group began to decrease from the third day. From the fourth day, cells in the observation group exhibited a relatively and obviously controlled growth, which showed that cells of the observation group significantly weakened their growth capacity. According to WHA and Matrigel penetrating test results, the migration velocity and the number of cells penetrating membrane/Matrigel in the observation group were obviously lesser than that in the blank group and empty vector group, which showed that the invasion and metastasis ability in the observation group was significantly lower than that of the latter two groups.

In summary, RUNX3 can inhibit the proliferation, migration, and invasion ability of human colorectal cancer HT-29, and its antineoplastic mechanism is through reducing the expression of MMP-2 and MMP-9, and ultimately inhibit the invasion and metastasis of colon cancer cells, but the pathway remains still unclear, which will also be our future research direction.

## Figures and Tables

**Table 1:** Invasion and metastasis of different HT-29 cells detected by the Transwell chamber and Matrigel invasion assay ($\bar{x}\pm s$).

Group	Number of migratory cells	Number of invasive cells
Blank group	201 ± 12	105 ± 10
Empty vector group	198 ± 11	107 ± 11
Observation group	73 ± 9^∗^	58 ± 7^∗^

^∗^ *P* < 0.05 compared with the control group.

## Data Availability

All data generated or analyzed during this study are included in this article.
